# A study of general practitioners’ perspectives on electronic medical records systems in NHSScotland

**DOI:** 10.1186/1472-6947-13-58

**Published:** 2013-05-21

**Authors:** Matt-Mouley Bouamrane, Frances S Mair

**Affiliations:** 1 University of Glasgow, College of Medical, Veterinary and Life Sciences, Institute of Health & Well-Being, Scotland, UK

**Keywords:** Medical informatics, Medical informatics applications, Information systems

## Abstract

**Background:**

Primary care doctors in NHSScotland have been using electronic medical records within their practices routinely for many years. The Scottish Health Executive eHealth strategy (2008-2011) has recently brought radical changes to the primary care computing landscape in Scotland: an information system (GPASS) which was provided free-of-charge by NHSScotland to a majority of GP practices has now been replaced by systems provided by two approved commercial providers. The transition to new electronic medical records had to be completed nationally across all health-boards by March 2012.

**Methods:**

We carried out 25 in-depth semi-structured interviews with primary care doctors to elucidate GPs’ perspectives on their practice information systems and collect more general information on management processes in the patient surgical pathway in NHSScotland. We undertook a thematic analysis of interviewees’ responses, using Normalisation Process Theory as the underpinning conceptual framework.

**Results:**

The majority of GPs’ interviewed considered that electronic medical records are an integral and essential element of their work during the consultation, playing a key role in facilitating integrated and continuity of care for patients and making clinical information more accessible. However, GPs expressed a number of reservations about various system functionalities – for example: in relation to usability, system navigation and information visualisation.

**Conclusion:**

Our study highlights that while electronic information systems are perceived as having important benefits, there remains substantial scope to improve GPs’ interaction and overall satisfaction with these systems. Iterative user-centred improvements combined with additional training in the use of technology would promote an increased understanding, familiarity and command of the range of functionalities of electronic medical records among primary care doctors.

## Introduction

The majority of healthcare encounters in the UK take place in a primary care setting, with a family doctor, commonly known as a General Practitioner (GP). GPs routinely use computerised systems within their practices. In Scotland, a majority of primary care practices used until recently, an Electronic Medical Records (EMR) system called GPASS (General Practice Administration System for Scotland). GPASS was initially developed in the mid-80’s and the system was provided to GPs free of charge by the NHS National Services Scotland [1]. The Scottish Health executive eHealth strategy (2008-2011) paved the way for the modernisation of the primary care electronic landscape in Scotland [2]. The procurement of primary care EMRs has since been delegated to the 14 territorial NHS boards of Scotland and the migration from GPASS to alternative accredited commercial systems had to be completed nationally by March 2012.

The introduction of new systems and technology is often seen to be disruptive in relation to existing practice [3]. Technology arrives with a set of assumptions about users’ needs, and these may not match user views and expectations. The latter may include views of appropriateness relative to the kind of data that technology might produce or transmit. Our previous experience suggests that eliciting user views may serve an important feedback agenda on the part of the users. As part of a study on information management processes in the patient surgical pathway in NHSScotland, we carried out 25 in-depth semi-structured interviews with primary care doctors between February 2012 and January 2013. This study examines the socio-technical factors that have influenced the adoption of electronic medical records systems within primary care practices in NHSScotland in order to inform future implementations in this sphere.

## Background & related work

### Primary care computing in NHSScotland

GPs are not employees of the National Health Service (NHS) but independent contractors and operate within their own premises (i.e *“a practice”*). They are responsible for dealing with the health needs of their registered populations which include all age groups. GPs provide community-based acute care, preventive care and have a key role in chronic disease management. In 2012, there were 4,859 practising GPs in Scotland clustered in 991 practices [4]. The majority of practices are operated by groups of GPs, effectively operating as small enterprises, employing nursing staff and healthcare assistants as well as a range of administrative support staff. The average number of patients registered with practices was estimated to be 5586 in October 2012 [5], but practice list sizes can range from several hundreds of patients to well over 20,000 for the largest practices in Scotland. Thus, GP practices require systems that allow them to coordinate the care of patients, by efficiently managing patients’ medical records, sharing information between treating GPs as well as transferring relevant information with other NHS care providers during the course of the patient treatment [6-8].

The General Medical Services (GMS) contract introduced in April 2004 a Quality & Outcomes Framework (QOF). GP practices are awarded points for meeting QOF targets, depending on the effective management of common chronic diseases, how well the practice is organised, patients’ experiences and a range of extra services which practices may provide. QOF measures achievement against a range of indicators, and additional payments to each practice are calculated based on performance in relation to these targets. QOF thus provides GPs with clear and defined financial incentives to record all healthcare episodes as accurately as possible [9,10].

### Impact of electronic medical records systems during the patient consultation

Greatbatch et al. studied the GP-patient interaction during consultations in a Liverpool practice, using a “before–after” comparison study design [11]. The study found that the introduction of desktop computer systems significantly impacted on the nature of the GP and patient communication behaviours. The following aspects of desktop computer use during the consultation were highlighted: the doctor using minimal verbal utterances while interacting with the computer, delaying responses until they had completed a task on the system, pausing while speaking to attend to the computer, focusing on the monitor or keyboard, gazing back and forth from the screen to the patient and abruptly changing topics to collect information required by the system. Patients for their parts often timed their speech utterances in order to avoid interrupting the doctor’s interaction with the system. The impact of information and communication technology (ICT) on doctor-patient communications during the consultation, often taking the place of a *“third party in the consultation”*, has also been highlighted in several recent studies [12,13]. Interestingly, several studies have also suggested that patients viewed the use of ICT by doctors during the course of the consultation as normal and that it did not negatively affect patient satisfaction [14,15]. In a survey of the perspectives of doctors and patients on information privacy in the EMR, Perera et al. found that – although patients had some reservations with regards the potential use of confidential information by third parties not directly involved in their care – they valued the potential benefits of electronic information sharing and aggregation when used specifically for their own health management [16]. Doctors also generally expressed positive opinions about EMR systems and had somehow less concerns regarding the potential risks to the privacy of patients’ medical information.

### Factors influencing adoption of electronic medical records systems in primary care

In a systematic review of factors promoting adoption of health information management systems (IMS), Ludwick & Doucette identified a range of factors which contributed to the outcomes of system implementation: user interface design, system functionalities, system suppliers, change and risk management processes, patient safety and quality of care, patient/doctor relationship, cost and systems effectiveness, training and users’ experience of technology [18]. Boonstra & Broekhuis also identified a wide range of potential barriers to EMR systems successful implementation and adoption which they categorised in eight main and inter-related categories: *‘financial, technical, time, psychological, social, legal, organizational, and change process’*[17]. They suggested that while some of these factors could potentially be addressed by system implementers, others were beyond their control, such as government sponsored financial incentives and privacy and data protection legislation and governance. In a systematic review of the impact of EMR systems on doctors’ practices, Lau et al. identified the following factors which influenced adoption: (i) technical design, performance and support affected usage and user satisfaction, (ii) implementation processes and workflows impacted on the practice productivity and coordination and (iii) performance-related financial incentives were important drivers for adoption [19]. In another systematic review of the impact of EMRs on structure, processes and outcomes, Holroyd- Leduc et al. suggested that EMRs seem to provide structural and process benefits in healthcare delivery (i.e. legibility, accessibility and perceived benefits on the quality of care) but that evidence on positive patient outcomes was lacking overall [20].

In a qualitative study of information management system impact on care coordination in the U.S., O’Malley et al. suggested that EMRs design was largely driven by documentation and billing rather than the needs of doctors and patients during the consultation [21]. Patient case management and collaborative decision-making remained difficult for health professionals, even when using the same EMR system. El-Kareh carried out a longitudinal study of primary care doctors’ perceptions of a new EMR system over a 12 months period and found that doctors’ satisfaction increased over time across a range of domains [22]. The number of doctors who felt that the new EMR system improved quality of care, reduced medication-related errors, improved follow-up of test results and communication among clinicians increased within one year of implementation. The number of those who felt that the EMR reduced the quality of patient interactions, increased the time spent on consultation and documentation tasks also decreased during the same period.

## Methods

### Data collection

Ethical approval for this study was obtained in February 2010 from the University of Glasgow College of Medicine, Veterinary and Life Sciences ethics committee. An invitation to participate in the study was sent to GP practices using a list compiled in April 2011 by the NHS Information Services Division [23]. We conducted 25 semi-structured interviews with GPs and 1 focus group between February 2012 and January 2013. The primary care practitioners sample target size initially set for this study was between 20 to 25 participants, and we ceased recruiting new GPs into the study once the upper limit was reached in January 2013. Interview duration ranged from half-an-hour to above an hour, with a mean duration of approximately 40 minutes per interview. The interviews were semi-structured and open-ended in order to allow the interviewer or interviewee to elaborate on unanticipated and potentially valuable information with additional questions, and probe for further explanation [24]. The interviews aimed to collect GP views on information management processes in the patient surgical pathway in NHSScotland: *information about the GP practice itself, including information management practices and ICT use, the patient consultation, referral processes to hospital outpatient clinics, communication between GPs and hospitals from the point of referral to patient surgery, post-operative discharge information provided by the hospitals, and finally, any issues identified in the patient surgical journey and areas for potential service improvement*[7,8,25,26].

19 interviews were conducted over the phone and 6 face-to-face. Interviews were recorded with participant consent and transcribed verbatim. Fifteen of the GPs were male and ten female. Most of the interviewees had been practicing GPs for a considerable number of years, with a range of 1 to 35 years and a mean of approximately 16.5 years. Respondents were from 9 of the 14 territorial health-boards of Scotland (*GP1–GP6: from NHS Greater Glasgow and Clyde, GP7–GP11: NHS Ayrshire & Arran, GP12 & GP13: NHS Dumfries & Galloway, GP14–GP16: NHS Fife, GP17: NHS Forth Valley, GP18: NHS Grampian, GP19–GP22: NHS Highlands, GP23: NHS Lanarkshire, GP24 & GP25: NHS Lothian)*.

### Data analysis

Interviews were analysed qualitatively using a thematic approach [24] and we then then used Normalisation Process Theory (NPT) as a conceptual framework to interpret the factors which were identified as facilitating or hindering the work of GPs during the patient consultation. An electronic health systems information management quality assessment framework was used for coding the transcripts [27]. The framework is derived from DeLone & McLean’s model of quality in information systems [28]. The framework comprises the following 6 dimensions: (i) eHealth information system quality, (ii) information quality, (iii) information usage, (iv) user satisfaction, (iv) individual impact and (vi) organisational impact. We also provide descriptive statistics and/or ratios where appropriate to illustrate how the range of perspectives expressed by each individual GP were representative of the overall sample of respondents.

NPT is concerned with the social organisation of the work (implementation) of making practices routine elements of everyday life (embedding) and of sustaining embedded practices in their social contexts (integration) and was developed particularly in response to the evidence, which suggested that eHealth implementation, embedding and integration are difficult to achieve in practice [29-31]. NPT aims to explain the routine embedding of practices by reference to the role of four generative mechanisms: *coherence; cognitive participation; collective action and reflexive monitoring*[3]. 

• **Coherence:** refers to the work of making a complex intervention hold together and cohere to its context, how people “make sense” or not of the new ways of working.

• **Cognitive participation:** is the work of engaging and legitimising a complex intervention, exploring whether participants buy into and/or sustain the intervention.

• **Collective action:** examines how innovations help or hinder professionals in performing various aspects of their work, issues of resource allocation, infrastructure and policy, how workload and training needs are affected and how the new practices affect confidence in the safety or security of new ways of working.

• **Reflexive monitoring:** is the work of understanding and evaluating a complex intervention in practice, and how individuals or groups come to decide whether the new ways of working are worth sustaining.

## Results: overall satisfaction with primary care electronic medical records systems

**Figure 1:**
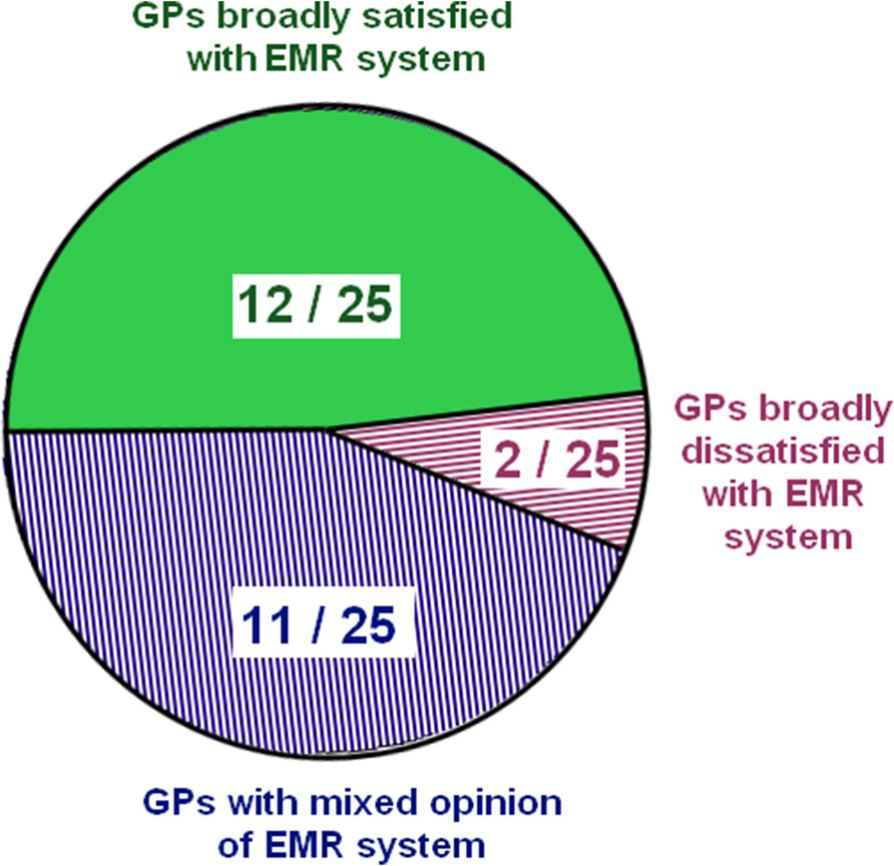
GP interviewees’ overall opinion on their electronic medical records system.

Two primary care EMR systems were used across all the practices surveyed: EMIS [32] and Vision [33]. 14 GPs reported using Vision in their practices and 11 reported using EMIS. The GPs reported having the system in use at their practice for just over 5 and a half years on average, with a range of 1 to 22 years. We asked interviewees to provide an overall opinion of their practice EMR system. We categorised responses in 3 groups: broadly satisfied, broadly dissatisfied and a mixed opinion (i.e. reporting some positive as well as negative aspects). n = 12/25 GPs (48%) reported an overall positive or very positive opinion of the practice EMR. n = 11/25 GPs (44%) expressed overall mixed feelings about the system. Finally, n = 2/25 GPs (8%) had an overall negative opinion of the EMR. The result of this overall impression is illustrated in Figure 1.

n = 11/25 GPs (44%) specifically mentioned functionalities which they thought were superior to some of GPASS: 

**GP21**:*“it’s been a stable system and easier to use then GPASS was, that’s why we chose it”*

n = 8 GPs (32%) mentioned that they felt that some (but not all) functionalities were better in the previous GPASS system, although that may have been in part due to the degree of familiarity the users had with the GPASS system: 

**GP15:***“it’s pros and cons... better it some ways than GPASS, GPASS had other things that was better about it so...”*

**GP20:***“We had GPASS before that and it worked fine for us, you know and we used it well but...* (about the new system) *we’ll get there...”*

A majority of users (n = 20/25, 80%) had switched to a new GP system within the last 6 years, including n = 11 (44%) who had only switched to new systems within the last 2 years. The average years of use for the group of GPs most satisfied with their systems was just over 7 and a half years. The average years of use for the GPs with a mixed opinion of their systems was lower at just over 4.5 years. 2 GPs who had expressed an overall negative opinion of the system reported using it for approximately one year and these users may have had additional difficulties in adapting to a new system compared to the other GPs we interviewed.

The trend of doctors’ increased satisfaction and decreased dissatisfaction over-time with the functionalities and impact of new EMR systems has also been reported in other studies [20,22]. 

**GP20**: *“I think, um... you’ve got 1 day training and I’m sure it does lots of lovely things but I’ve not idea how to do them... I mean, I can print... add the odd diagnosis but it’s... if you don’t know the actual work that they want, it is really difficult...”*

*“I can print prescriptions... but it’s... anyway... it’s just... you try to find time to do a bit of it but I... you know... I suppose I need to sit with somebody who is using it all the time and using it well because I’m sure that there’s a lot of things that you can do with it but I’m still struggling a year on...”***GP24**:*“GPASS was very useful, very good, and I’m very sorry to see the back of it”*

(asked what could improve the current EMR:)

“I think if we could refund GPASS and start again with that”

There was no immediate association between GPs’ years of practice and levels of satisfaction with the practice EMR, as has been reported elsewhere [34]. Indeed, the following comment from a GP with 30 years of practice helps to illustrate the latter point: 

**GP22:***“...I am certainly old-fashioned and reactionary but I am not blind to the huge benefits [...] if I were to sit down and list all the benefits of the computing system, I could make a huge list...”*

## Thematic evaluation of primary care electronic medical records systems

After this brief overview of GPs’ overall satisfaction levels with their EMR system, we invited respondents to further elaborate on any specific aspect of the systems that they perceived as useful or else, cumbersome or unhelpful. GP’s responses are here presented in the following 3 thematic dyads, using the eHealth system quality framework derived from DeLone & McLean’s model of information systems’ quality [27,28]:

(i) information system and information quality

(ii) information usage and user satisfaction

(iii) individual and organisational impact

### EMR & Information quality

#### Perceived benefits of information systems

n = 18 out of 25 GPs spontaneously reported some perceived benefits with their EMR, including the following features: 

- the EMR provides adequate support for information access and searching, (n = 13/25)

- the EMR technology is up to date, stable and reliable, and functionalities are superior to that of previous systems, (n = 11/25)

- the EMR is flexible, adaptable, with a broad range of functionalities and provides adequate work-flow support, (n = 10/25)

- the EMR provides adequate support for data entry, clinical coding and record keeping, (n = 9/25)

- the EMR supports well electronic prescribing, (n = 3/25)

#### Improved access to information

As one would perhaps expect, the GPs found improved access to patient medical information one of the main advantages of the practice EMR, including convenient access to the patient record, access to patient medical summaries, the ability to filter information based on a specific diagnosis or medication and access to immunisation data:

**GP3**:*“... you can pull up things like summaries of results much easier [...] You can do searches and things a lot easier. So there’s a lot of advantages...”*

**GP9**: *“... The fact that it reads most things that you record. So everything is searchable. And the fact that you can search it, so you can search by keyword through the entire patient record which is very useful....”*

**GP23**:*“...It’s very easy to do searches: if you wanted to find someone who the last time they came up with – say: a sore elbow – you just put ‘elbow’ into the search bar and it will throw you up all the consultations where they mentioned elbow.”*

#### Perceived Dis-benefits of information systems

n = 13 out of 25 GPs spontaneously reported some perceived flaws with their EMR, including the following features: 

- the EMR is administratively cumbersome and/or not sufficiently flexible to support workflows, (n = 7/25)

- the electronic prescribing functionalities are not optimum to support existing work-practices, (n = 7/25)

- occasional system breakdown compromises work practices on the day of system failure, (n = 3/25)

System failures were reported as infrequent but caused substantial disruption to patient consultation when they did occur: 

**GP12:***“... It’s not a great feeling when you go down on a Monday morning and [...] the practice manager [...] says: ‘the computers aren’t working this morning and we haven’t got a clue who’s going to turn up’ [...] I remember one Monday morning we had about two patients that had come back to discuss results and I had... just had to say: ‘I’m sorry the computer’s down you have to go and make another appointment.’ ”*

**GP13:***“well, the main draw-back... [...] there were days when the system crashed... and in that case, what... because you’re so reliant on this, you would end-up seeing patients and saying ‘I’m sorry. Huh, but I don’t have access to your old record, so we will just have to, you know... huh, go by what you say and what you recall, humh...’ the dynamics of the GP consultation is – with most patients who are attending frequently – they would assume that the doctor would have access to their record and when they come in and, for a 10 minutes consultation, umh... you know, they wouldn’t expect that they would have to recall all their events and all their history so... if the system crash, you’re kind of, huh... you’ve got no back-up”*

Both EMIS and INPS provide streaming solutions (EMIS Web [35] and Vision 360 [36]) so that copies of clinical records held on local GP systems can be stored online on remote servers, thereby providing back-up access in the event of local system failure. However, while these additional online back-up solutions have been purchased and provided by a number of health-boards, they are not currently available to all GP practices across Scotland.

### Information usage & user satisfaction

#### Perceived benefits covered four main areas

- the EMR provides useful information added value, include key-work based searches, information filtering, clinical summaries, and features for classification and categorisation, (n = 10/25)

- the EMR provides useful decision support features, (n = 5/25)

- training and experience allowed GPs to use the system with confidence, (n = 5/25)

- the EMR includes features which supports information sharing with patients, (n = 2/25)

#### Improved patient safety feature

Several GPs considered the fact that the practice EMR only allowed the user to have a single patient record opened at any one time as an improved and important embedded patient safety feature. The previous system allowed users to open multiple records concurrently, which increased the risk of mistakenly entering data in the wrong patient record. Also, the new systems include a number of decision support functionalities such as alerts and reminders. 5 GPs specifically mentioned decision support as a useful feature of the systems. 

**GP19**:*“... I think the sort of alerts, the clinical alerts, you know, it’s sort of got an in-built system where it will flash up pointers, you know: “this patient’s blood pressure needs taken”, or it links in with the Quality Outcomes Framework system for general practice... it gives you a reminder, instant reminders...”*

#### Perceived dis-benefits

- the EMR has some information navigation issues and unnecessary steps (e.g. multiple clicks), (n = 9/25)

- a lack of training and understanding of the system prevents the GPs to use the system to its full potential, (n = 5/25)

#### Usability and navigation issues

Several GPs encountered usability issues when using the system : 

**GP7**: *“... it tends to be... to involve quite a lot of clicking and I’d quite like to have it simplified in using fewer steps. So... but that’s about all. I am really genuinely fairly happy with it”*

“There are things that you could... you could do in one mouse click or one key-stroke that take 3 or 4. And by the time you’ve multiplied that by doing it a hundred times, it’s an awful lot of extra key-strokes. And that’s my main gripe about it, there seems to be a lot of unnecessary steps that if somebody just went to (see) how you use it, we’d be able to cut that down...”

**GP10**:*“...I maybe don’t know the easiest ways of doing things. Or you do something for years and then you discover that there’s a much easier way of doing it that everybody else seems to know and you didn’t. For no good reason.”*

**GP18**:*“It’s a quite a busy front page and there’s many ways of accessing information... different routes into it which is quite complex”*

**GP22**:*“...personally, I am finding it difficult to teach myself exactly where everything is and how to transfer from one screen to another”*

**GP23**:*“...multiple clicks, it’s not very good, not user-friendly. Sometimes it’s a bit too much on the screen, it’s multiple screens, it’s hard to really see where’s the flow of the consultation [...] (an improvement would be)... if you could use the keyboard more than this multiple click thing”*

#### Information and alerting overload

Several GPs raised information overload, particularly on the screen estate as a hindrance to the consultation: 

**GP1**:*“The acute stuff can be a bit tedious because of all the warnings it now comes up (with). And there’s so many warnings that one just... You can’t see the wood for the trees, but I suppose it could help there if there’s a bit more time (during the patient consultation)”*

**GP2**:*“... The main drawback is on the... we get our results direct... in the general screen, which is actually quite a useful screen, which is everything in chronological order. Since we’ve got the results direct into that screen, each result is on a separate line so there’s... that screen’s really kind of clogged up with stuff and you can’t see easily. And it’s also quite hard to because of the way the results are presented on the screen, it’s actually quite hard too to read.”*

**GP19**:*“the screen is very busy, you know the screen is extremely busy”*

#### Difficulties in adapting to the new system

As previously suggested, several GPs had made a relatively recent switch to a new system based on NHS requirements. Several had become accustomed to the previous system and found it difficult to adjust to the new system: 

**GP22**:*“I don’t enjoy computer work [...] with me, it was a case of ‘better the devil you know’ [...] I am taking the responsibility that’s my inadequacy rather then the system inadequacy [...] there are things I have difficulties with but I don’t necessarily blame the system for that”*

**GP23**:*“There’s a lot of things you can do with it, but it’s also very, very time consuming.”*

### Individual & Organisational impact

#### Perceived positive impacts

- the EMR provides good support for record-keeping, access, retention and performance monitoring in the practice, (n = 11/25)

- the introduction of the EMR has positive impact on the office space, work environment (i.e. by reducing the use of paper records and forms across the office and reducing storage needs), (n = 7/25)

- the introduction of the EMR leads to individual and collective improvement in effectiveness and performance in the practice, (n = 6/25)

#### Improved audit

The GMS GP contract entails providing performance data to the health services and the practice EMR was perceived as indispensible for these tasks: 

**GP1**: *“general practice is driven by its contract and the contract is only operational because of the IT system... it relies on measuring, that’s what IT systems do so well and without that, we couldn’t do it”*,

**GP16**:*“you could not do the job of following the GP contract now without computer... it’s not as if you have a choice not to have computers... they are part of the job”*,

**GP22**: *“I think there are huge benefits, certainly from the point of view of auditing service provision, it makes a lot of sense...”*,

**GP23**:*“... It’s very good to search with and there are different ways of doing things with it so, audit-wise, it’s very good ”*

#### Impact of EMR on record-keeping

In 2003, a study by Morris et al. found that a large majority of GPs (94%) routinely used computer systems in the course or their duties but only 3% of practices surveyed at the time reported being entirely paperless [37]. However, a more recent report by the British Medical Association reported that 90% of practices in Scotland were either paper-less or “paper-light” [9].

Using electronic patient records had a substantial impact on work processes within the practice, both in terms of a reduced burden on administrative staff, and the reduced physical area required for storage of legacy paper records. It also means that the nature of the work of administrative staff, and thus their skill requirements are evolving with the routine use (embedding) of computerised systems: 

**GP3**: *“it would be nice to completely get rid of the paper notes and have them all in... you know, have them all online in the patient’s (system)... but that would take so much time and effort (i.e. to scan paper documents in the system), it’s probably not worth it really. Because these notes... as every year passes, these notes become less and relevant... [...]*

“The girls are losing their skills in finding them (the patient paper records). And some of them have never really used them much, so they find it more difficult. Some of the others were well used to it, so they have still got their skills [...] But we’re using them less and less as it goes further on, the more and more we have in the electronic (records) and the better that is.”

**GP7**: *“I mean it’s very much less manpower intensive not using paper records”*,

**GP12**: *“I think generally being paperless is better, and all the information’s is on the computer, so you can get hold of stuff”*, *“...sometimes you can get hold of other records more quickly. I think it frees up receptionist time from filing and all that sort of stuff...”“...the receptionist staff [...] even like pulling all the notes for all the, you know, appointments and stuff, and then having to file them away, I mean I think that saves them probably a good hour, maybe two hours, a day [...] I think it certainly makes their job a bit better, it does.”*,

**GP14**: *“You don’t lose things so much now [...] It probably does reduce our receptionist time from hunting bits of paper, paper records and also now that all the blood results all just come through online, so we don’t have to fill in all these bits of papers as well...”*,

**GP17**: *“we started scanning in everything about 8 years ago and then we started... We kept paper records up until 3 years ago and then we’ve – you know, just as a back up – but we’ve now been able to shred. If we get paper records, we scan them and then we just shred the paper records...”*,

**GP22**: *“as far as staffing is concerned too, the fact that – physically – not having to find (paper) notes back and forward... their jobs’ description is going to change quite dramatically and... computer literacy for my staff obviously has to be... well... they’re ahead of me, I have to say...”*

#### Facilitator of shared-care

Several GPs suggested that having an online patient record facilitated shared and continuity of care: 

**GP12**: *“ in the old days if you had, sort of like, one set of notes or something, sometimes like maybe the nurses want to look up something about a patient or something and if you had the notes, it was kind of awkward. Whereas now they could more or less look at the notes at the same time...”*,

**GP17**:*“it’s one clinical record that any, the staff that work in the practice can input information into, so we’ve got our district nurses put their information in and the practice nurses put their information in... so it’s a shared clinical record. You don’t... you can’t lose it and it means that more than one person access it at any time, so it’s not like, you know... one person’s got the notes and nobody else can see them”*

#### Perceived negative impacts

- there is insufficient organisational support or resources (e.g. from the health-board) to support the training of staff and deployment of new ICT systems, (n = 3/25)

- the EMR is not sufficiently integrated with other electronic systems used in the practice, (n = 2/25)

- the introduction of ICT is having a negative impact on existing work practices, (n = 2/25)

- the introduction of ICT is having a negative impact on the clinical encounter, (n = 1/25)

#### Issues of interoperability of systems and systems integration

Several GPs found switching between several systems cumbersome : 

**GP9**:*“... Disadvantages are having to flick from one system to another, so you have to flick from Vision to Docman, to mail manager... it isn’t all there [...] So there are three separate systems that run that don’t automatically, I mean they do coordinate a bit, but they’re not perfect [...] it’s slow, you have to log in separately, it takes the computer some time.”*

**GP10**:*“... I’m afraid I have little understanding of it all, I do as I’m told. But you know if you have a patient record open and you’re looking for results that have been done at the hospital, you can’t access it from the patient record, you have to open the internet and then go into through the SCI (Scottish Care Information Gateway) and find out that way and type it all, it’s cumbersome.”*

#### Interference in the patient consultation

One GP had very strong views about how ICT systems and performance-driven work practices compromised the patient-doctor relationship: 

**GP1**:*“it’s like having a third person in the room so it’s quite... It can be quite disruptive in consultation as well.”*

“[...] Well both parties, both me and the patient can find their eyes drawn to the screen. That’s not really, I mean it’s a bit like having a conversation with somebody with the TV on in the background.”

“the IT revolution is destroying what was great about British general practice...” [...] “the stuff that we can’t measure – like the human compassion side of health – is being squeezed out by the need to record frequently meaningless data”

“ [...] nobody gives me any extra money for, you know, giving some patient a hug, the cuddle factor doesn’t attract QOF points”

This last comment echoes the concerns of a previous study which cautioned that financially incentivised performance targets strongly shaped the roles of primary care teams and the nature of activities, with less attention and efforts being allocated to non-incentivised activities [38]. This should also be seen in the light of a recent systematic review on the impact of the QOF in the UK which found modest improvements in quality of care for chronic diseases and an uncertain impact on costs, professional behaviour, and patients’ experiences [39].

## Interpretation & discussion

Using the 4 NPT constructs, we review and interpret the findings of our study in turn:

### Coherence: *‘Making sense of new electronic systems’*

It is clear that considerable effort has been put into policy building and dissemination of information both locally and nationally in relation to the universal switch to a new primary care EMR in March 2012. The GPASS system was until relatively recently used by around 80% of practices in Scotland [40,41]. However, many GPs increasingly felt that the system was no longer meeting their needs. The Scottish Local Medical Committee Conference (2006) called for GPASS to be replaced by alternative systems [9]. EMR systems have been in wide-spread use in Scotland for many years and their adoption is now – to the best of our knowledge – almost, if not entirely universal [42]. A majority of GPs interviewed considered that their EMR system was to some degree beneficial to their work practices. Most stakeholders were clear about the need for change and this has facilitated the development of *coherence*, that is, a shared view of the purpose of these initiatives, with individuals able to grasp potential benefits and has facilitated normalisation of these new technologies. The key lesson here is that the successful adoption of new technology therefore needs to be seen as the result of a sustained effort to communicate the rationale for change and sustained efforts to promote changes in practices, culture and IT use within NHSScotland over a prolonged period.

### Cognitive participation: *‘Achieving buy-in’*

Although the work of engaging with users is central to the successful implementation of any new technology, work aimed at actively involving GPs in the take-up of new EMRs was barely mentioned in the interviews. While many GPs felt that the previous GPASS system was no longer fit for purpose, most had been using it for years. Many felt that it was – although perhaps not optimum – a system that they had grown familiar with and felt confident using. Several GPs felt that they had received insufficient training before having to switch to the new EMR systems within their practices. However, both INPS and EMIS vendors have provided individualised progressive migration calendars to primary care practices, including training sessions during systems transition and several of the GPs we interviewed also admitted that they too had some responsibility towards making the effort required to improving their skills with the new system. However, they often cited a lack of time as a barrier to do so. The considerable time and effort required to adopt new electronic primary care systems has also been reported in other studies, which suggested that dedicated time for training as well as basic knowledge of ICT were important factors in the successful adoption of these systems [43].

A substantial incentive for the use of practice EMR was audit-related tasks to implement the QOF and this was a key feature. Also, the visible benefits, for example, in terms of improved access to patient information was clearly a positive driver to uptake. However, it is clear that – although there may have been deficiencies in some aspects of the system functionalities – the presence of financial incentives and other system benefits outweighed the barriers to the uptake and adoption of the new systems. Performance-related financial incentives were also identified as important drivers of EMR adoption in a systematic review of the impact of EMR systems in primary care practices [19].

### Collective action: *‘Operationalising new systems’*

The emphasis of collective action involves the work performed by individuals, groups of professionals or organisations in operationalising a new technology in practice and socio-technical issues, such as how e-health systems affected the everyday work of individuals, organizational structures and goals [3]. The impacts of practice EMR in that respect are substantial. Overall it is clear that the uptake, adoption and normalisation of these new systems have been possible because, to a large extent, they make the completion of clinical tasks easier.

While GPs will usually work alone during the patient consultation and interact individually with the EMR, an electronic repository of clinical records will facilitate the sharing of patient data with other health professionals within the practice (i.e. nurses and other GPs) and within the health-boards (i.e. with district nurses), as well as enabling electronic transfer of patient information to secondary care services through electronic referrals [8]. This was considered by a majority of GPs as an important step towards an integrated patient care pathway within the NHS [7].

Several GPs considered that the EMR was therefore a facilitator of shared and continuity of care. Many GPs work part-time and the EMR enables the treating GP to have immediate access to a patient record which may have been accessed and modified by another member of staff within the practice. It also allows for GPs, nurses and healthcare assistants to have concurrent access to the patient medical record. Within the practice, the EMR integrates with an electronic document repository (Docman), allowing to store patient laboratory results and clinical letters such as hospital discharge information. The EMR records are also used to transfer information to the local health-boards electronic data repositories (SCI Store). This information is used among other purposes to populate the patient Emergency Care Summary, available in secondary care hospitals in case of clinical emergencies [6].

#### Impact on workflows

Many GPs reported perceived usability issues with their EMR and several attributed this to a lack of understanding of their work by system developers. However, the EMR is a complex artefact and it is not entirely clear how individual tasks and functionalities could be further simplified in future. In addition, it is likely that some of the perceived difficulties GPs have with their systems could actually be resolved through additional training and familiarisation with the systems. Indeed, our results suggest an increased overall satisfaction with the EMR systems according to the length of use, which has also been reported in other studies [20,22]. The use of EMR also had a substantial impact within the broader practice, in terms of space and storage. As a consequence, administrative support tasks within the practice are now heavily reliant on the use of ICT: for booking patient appointments, record-keeping, quality assurance of clinical coding and completing electronic referral letters on behalf of GPs [8].

Recurrent usability issues during the course of the consultation, such as ‘multiple clicks’ – often perceived by GPs as frustrating and unnecessary – have frequently been reported. The format of our study can not ascertain whether these were legitimate usability issues or else, embedded checks and safety features which were not perceived as such by GPs. In any case, it appears that this potential distinction was not clear to end-users. Furthermore, this also suggests that the use of ‘multiple clicks’ as an error prevention mechanism can be perceived as a blunt instrument for avoiding clinical errors in EMR systems, particularly if this feature is recurrent throughout the system. While it might make sense from the system developers’ point-of-view to introduce double-checks at key decision points – as a typical consultation will usually last approximately 10 minutes on average – the frequency of this type of system interaction can be very high (i.e dozens or even hundreds of times a day), therefore becoming disproportionally frustrating for GPs in the course of the consultation.

#### Roles, responsibilities and training

The routine use of EMR has an impact on medical training as recently qualified GPs had all trained with one or several emr systems and consequently appeared more comfortable in using or switching from one system to another. Yet, even recently qualified GPs had some difficulties and reservations when using their practice EMR which raises the question of whether further ICT training would be a useful addition to their medical training?

The lack of ICT skills among GPs has been identified as a safety concern in other studies. A previous study by Morris et al. suggested that – although GPs in primary care trusts in England ranked patient safety highly – they often had insufficient knowledge and training to make optimum use of embedded clinical decision support features of their computer systems [44]. Shojania et al. suggested in their systematic review on the impact of computer decision support systems (CDSS) on doctors’ behaviour that computer reminders only provided modest improvements on clinical processes and guideline adherence [45]. Avery et al. conducted semi-structured interviews with a range of key stakeholders of GP computer systems in order to identify features which could lead to patient safety improvement, particularly in the area of medication prescribing and decision support alerting [46]. The authors suggested that a concerted effort from a range of stakeholders would be needed to promote increased safety in the use of ICT in primary care. This would include: additional training of primary care practitioners in the effective use of ICT systems, incentives for systems developers to improve the safety features of their systems and the importance of change management to promote an increased use of ICT for safety purposes. Short et al. identified a number of barriers to the use of CDSS in general practice consultations, including: limited time and consequently the potentially infrequent use of a CDSS, GPs’ limited skills in ICT, a lack of understanding and the risk assessment functionalities, algorithms and results, the reluctance of GPs to rely on a third-party system for risk assessment, the potential concerns of patients with a CDSS and the possible lack of patients’ understanding of risk results [47].

### Reflexive monitoring

Reflexive monitoring deals with the evaluation and monitoring of eHealth implementations and how these are used to influence utilisation and future implementations [3]. There was little evidence in the interviews of local appraisals of the ways in which implementation processes or EMR systems might be reconfigured by user-produced knowledge. Both GP system vendors provide online support for their community of users in NHSScotland. In addition, there also seems to be some local support available at the health-board level: both at the time of system transition and on an ongoing-basis, with regards collecting system specifications and change requirements from the local GP practices. However, there could clearly be the potential for substantial benefits, for example, if a majority of GPs were to become more proactive in communicating usability and functionality concerns to system developers.

## Conclusion

This study is the first to collect GPs’ perspectives at an important transition point in the primary care computing landscape in Scotland. ICT implementations in healthcare delivery systems are complex and influenced by a range of factors at individual and organisational levels. Monitoring system use in the early stages of implementation is essential to understand the factors promoting adoption [3,48].

Primary care doctors play a central role in health service delivery and thus, it is essential to conduct studies which elucidate an understanding of their opinions, perspectives and work processes. EMR systems are now essential to assist GPs and practice staff to carry out their duties, including: patient care, record-keeping, auditing and information transfer to other care providers within the broader national health system. GPs consider electronic information systems as a mean to an end: that of patient care and practice management. While the majority of GPs considered that EMR systems provided important benefits, our study also highlights that there remains substantial scope to improve GPs’ interaction and overall satisfaction with these systems. Iterative user-centred system improvements, combined with additional training in the use of technology, would allow primary care doctors to gain an increased understanding, familiarity and command of the range of EMR system functionalities.

## Abbreviations

CDSS: Computer clinical decision support system; EMR: Electronic medical records system; GMS: General medical services; GP: General practitioner; GPASS: General practice administration system for Scotland; ICT: Information & communication technology; NHSScotland: National health service for Scotland; NPT: Normalisation process theory; QOF: Quality & outcomes framework; SCI: Scottish care information group

## Competing interests

The authors declare that there are no conflicts of interest.

## Authors’ contributions

M-MB and FSM conceptualized the project. MMB analysed the data, performed the literature and internet searches and drafted the manuscript. FSM critically reviewed and revised iterative versions of the drafted manuscripts. All authors read and approved the final manuscript.

## Pre-publication history

The pre-publication history for this paper can be accessed here:

http://www.biomedcentral.com/1472-6947/13/58/prepub

## References

[B1] http://www.nhsnss.org/

[B2] The Scottish Government eHealth Strategy 2008–2011 2008 24

[B3] Mair FS May C O’Donnell C Finch T Sullivan F Murray E Factors that promote or inhibit the implementation of e-health systems: an explanatory systematic review J Bull World Health Organ 2012 90 357 364 10.2471/BLT.11.099424 PMC3341692 22589569

[B4] Information Services, Division - ISD Scotland / NHS National Services Scotland General practice – GP workforce and practice population statistics to 2012 Nat Stat Publication Scotland 2012 12 [ http://www.isdscotland.org/Health-Topics/general-practice/Publications/]

[B5] http://www.isdscotland.org/Health-Topics/General-Practice/Practices-and-Their-Populations/

[B6] Bouamrane MM Mair F An overview of electronic health systems development and integration in Scotland Proceedings of Managing Interoperability and Complexity in Health Systems, MIXHS’11, 20th ACM Conference on Information and Knowledge Management, CIKM 2011, Glasgow, U.K., October 24-28, 2011 2011 New York, NY, USA: ACM 59 62

[B7] Bouamrane MM Mair F A study of Information Management in the Patient Surgical Pathway in NHSScotland Proceedings of the 14th World Congress on Medical and Health Informatics, Medinfo 2013 2013 Copenhagen, Denmark: IOS Press in press 23920617

[B8] Bouamrane MM Mair F Evaluation of a nation-wide protocol-driven electronic referral system J Under Peer-Rev 2013

[B9] Scotland BMA General Practice in Scotland: The Way Ahead - Final Report BMA Scotland, British Medical Association; 2010:20

[B10] Williams T van Staa T Puri S Eaton S Recent advances in the utility and use of the general practice research database as an example of a UK primary care data resource J Ther Adv Drug Saf 2012 3 2 89 99 10.1177/2042098611435911 PMC4110844 25083228

[B11] Greatbatch D Heath C Campion P Luff P How do desk-top computers affect the doctor-patient interaction? J Fam Pract 1995 12 1 32 36 10.1093/fampra/12.1.32 7665038

[B12] Gibbings-Isaac D Iqbal M Tahir MA Kumarapeli P de Lusignan S The pattern of silent time in the clinical consultation: an observational multichannel video study J Fam Pract 2012 29 5 616 621 10.1093/fampra/cms001 22291439

[B13] Pearce C Arnold M Phillips C Trumble S Dwan K The patient and the computer in the primary care consultation J JAMIA, J Am Med Inform Assoc 2011 18 2 138 142 10.1136/jamia.2010.006486 PMC3116262 21262923

[B14] Lelievre S Schultz K Does computer use in patient-physician encounters influence patient satisfaction? J Can Fam Physician 2010 56 1 e6 e12 PMC2809189 20090064

[B15] Ridsdale L Hudd S Computers in the consultation: the patient’s view J Br J Gen Pract 1994 44 385 367 369 PMC1238955 8068397

[B16] Perera G Holbrook A Thabane L Foster G Willison DJ Views on health information sharing and privacy from primary care practices using electronic medical records Int J Med Inform 2011 80 2 94 101 10.1016/j.ijmedinf.2010.11.005 21167771

[B17] Boonstra A Broekhuis M Barriers to the acceptance of electronic medical records by physicians from systematic review to taxonomy and interventions BMC Health Services Res 2010 10 1 231 10.1186/1472-6963-10-231 PMC2924334 20691097

[B18] Ludwick D Doucette J Adopting electronic medical records in primary care Lessons learned from health information systems implementation experience in seven countries Int J Med Inform 2009 78 1 22 31 10.1016/j.ijmedinf.2008.06.005 18644745

[B19] Lau F Price M Boyd J Partridge C Bell H Raworth R Impact of electronic medical record on physician practice in office settings: a systematic review BMC Med Inform and Decis Mak 2012 12 1 10 10.1186/1472-6947-12-10 22364529 PMC3315440

[B20] Holroyd-Leduc JM Lorenzetti D Straus SE Sykes L Quan H The impact of the electronic medical record on structure, process, and outcomes within primary care: a systematic review of the evidence J Am Med Inform Assoc 2011 18 6 732 737 10.1136/amiajnl-2010-000019 21659445 PMC3197985

[B21] O’Malley AS Grossman JM Cohen GR Kemper NM Pham HH Are electronic medical records helpful for care coordination? Experiences of physician practices J Gen Intern Med 2010 25 3 177 185 10.1007/s11606-009-1195-2 20033621 PMC2839331

[B22] El-Kareh R Gandhi TK Poon EG Newmark LP Ungar J Lipsitz S Sequist TD Trends in primary care clinician perceptions of a new electronic health record J Gen Intern Med 2009 24 4 464 468 10.1007/s11606-009-0906-z 19156468 PMC2659149

[B23] http://www.isdscotland.org/

[B24] Kaplan B Maxwell J Anderson J, Aydin C Qualitative Research Methods for Evaluating Computer Information Systems Evaluating the Organizational Impact of Healthcare Information Systems, chapter 2. Health Informatics 2005 New York: Springer 30 55

[B25] Bouamrane MM Mair F Managing complexity in pre-operative information management systems Proceedings of Managing Interoperability and Complexity in Health Systems, MIXHS’11, 20th ACM Conference on Information and Knowledge Management, CIKM 2011, Glasgow, United Kingdom, October 24-28, 2011 2011 New York, NY, USA: ACM 3 10

[B26] Bouamrane MM McGee-Lennon M Brewster S Mair F Using process-mapping to design integrated health information management systems Computer-Based Medical Systems (CBMS), 2011, 24th International Symposium on 2011 Washington, DC, USA: IEEE Computer Society 1 6

[B27] Bouamrane MM Tao C Mair FS An Overview of Electronic Health Information Management Systems Quality Assessment Proceedings of Managing Interoperability and compleXity in Health Systems, MIXHS’2012, 21st ACM International Conference on Information and Knowledge Management, CIKM 2012, Maui, USA 2012 New York, NY, USA: ACM 37 46

[B28] Delone WH McLean ER Information systems success: The quest for the dependant variable Inform Syst Res 1992 3 1 60 95 10.1287/isre.3.1.60

[B29] May C Mair F Finch T MacFarlane A Dowrick C Treweek S Rapley T Ballini L Ong BN Rogers A Murray E Elwyn G Gunn J Montori VM Légaré F Development of a theory of implementation and integration: Normalization Process Theory Implementation Sci 2009 4 29 9 10.1186/1748-5908-4-29 PMC2693517 19460163

[B30] May C Finch T Implementing, embedding, and integrating practices an outline of normalization process theory Sociology 2009 43 535 554

[B31] Bouamrane MM Osbourne J Mair F Understanding the implementation and integration of remote tele-health services; an overview of normalization process theory Pervasive Computing Technologies for Healthcare (PervasiveHealth), 2011 5th International, Conference on 2011 DC, USA: IEEE Computer Society, Washington 300 307

[B32] EMIS™ http://www.emis-online.com/

[B33] INPS™ http://www.inps4.co.uk/

[B34] Simon SR Kaushal R Cleary PD Jenter CA Volk LA Orav EJ Burdick E Poon EG Bates DW Physicians and electronic health records: A statewide survey Archives of Internal Med 2007 167 5 507 512 10.1001/archinte.167.5.507 17353500

[B35] EMIS Web for GPs http://www.emis-online.com/emis-web-for-gps

[B36] Vision 360 http://www.inps4.co.uk/vision360/

[B37] Morris L Dumville J Campbell LM Sullivan F A survey of computer use in Scottish primary care general practitioners are no longer technophobic but other primary care staff need better computer access Inform Prim Care 2003 11 1 5 11 16274587 10.14236/jhi.v11i1.550

[B38] Maisey S Steel N Marsh R Gillam S Fleetcroft R Howe A Effects of payment for performance in primary care: qualitative interview study J Health Services Res & Policy 2008 13 3 133 139 10.1258/jhsrp.2008.007118 18573761

[B39] Gillam SJ Siriwardena AN Steel N Pay-for-performance in the United Kingdom: impact of the quality and outcomes framework – A systematic review Ann Fam Med 2012 10 5 461 468 10.1370/afm.1377 22966110 PMC3438214

[B40] Milne RM Taylor MW Taylor RJ Audit of populations in general practice: The creation of a national resource for the study of morbidity in scottish general practice J Epidemiol Commun Health 1998 52 Suppl 1 20S 24S 9764266

[B41] NHS National Services Scotland Supporting Scotland’s health... every day. NHS National Services Scotland annual report 2008/2009 2009 50

[B42] Protti D Wright G Treweek S Johansen I Primary care computing in England and Scotland: a comparison with Denmark Inform Prim Care 2006 14 2 93 99 17059698 10.14236/jhi.v14i2.619

[B43] Terry AL Thorpe CF Giles G Brown JB Harris SB Reid GJ Thind A Stewart M Implementing electronic health records. Key factors in primary care Can Fam Physician 2008 54 5 730 736 18474707 PMC2377228

[B44] Morris CJ Savelyich BSP Avery AJ Cantrill JA Sheikh A Patient safety features of clinical computer systems: questionnaire survey of GP views Qual Saf Health Care 2005 14 3 164 168 10.1136/qshc.2004.011866 15933310 PMC1744017

[B45] Shojania K Jennings A Mayhew A Ramsay C Eccles M Grimshaw J Effect of point-of-care computer reminders on physician behaviour: a systematic review CMAJ 2010 182 5 E216 25 10.1503/cmaj.090578 20212028 PMC2842864

[B46] Avery AJ Savelyich BSP Sheikh A Morris CJ Bowler I Teasdale S Improving general practice computer systems for patient safety: qualitative study of key stakeholders Qual Saf Health Care 2007 16 1 28 33 10.1136/qshc.2006.018192 17301200 PMC2464931

[B47] Short D Frischer M Bashford J Barriers to the adoption of computerised decision support systems in general practice consultations: a qualitative study of GPs’ perspectives Int J Med Inform 2004 73 4 357 362 10.1016/j.ijmedinf.2004.02.001 15135754

[B48] Gagnon MP Desmartis M Labrecque M Car J Pagliari C Pluye P Frémont P Gagnon J Tremblay N Légaré F Systematic review of factors influencing the adoption of information and communication technologies by healthcare professionals J Med Syst 2012 36 241 277 10.1007/s10916-010-9473-4 20703721 PMC4011799

